# One-Step Multiplex Real-Time Fluorescent Quantitative Reverse Transcription PCR for Simultaneous Detection of Four Waterfowl Viruses

**DOI:** 10.3390/microorganisms12122423

**Published:** 2024-11-25

**Authors:** Chenchen Wang, Huixin Liu, Junze Cheng, Sijia Pan, Wenwen Yang, Xiaofang Wei, Yue Cheng, Ting Xu, Hongbin Si

**Affiliations:** Guangxi Key Laboratory of Animal Breeding, Disease Control and Prevention, College of Animal Science and Technology, Guangxi Grass Station, Guangxi University, Nanning 530004, China; wycx99903724@sina.com (C.W.); 13317815805@163.com (H.L.); 13525862539@163.com (J.C.); p18177273738@163.com (S.P.); mqxshawan@163.com (W.Y.); 2218402005@st.gxu.edu.cn (X.W.); cy18071728295@163.com (Y.C.); 18345760639@163.com (T.X.)

**Keywords:** duck Tembusu virus, duck hepatitis virus, Muscovy duck reovirus, Muscovy duck parvovirus, real-time fluorescent quantitative reverse transcription PCR

## Abstract

Duck Tembusu virus (DTMUV), duck hepatitis virus (DHV), Muscovy duck reovirus (MDRV), and Muscovy duck parvovirus (MDPV) represent four emergent infectious diseases impacting waterfowl, which can be challenging to differentiate due to overlapping clinical signs. In response to this, we have developed a one-step multiplex real-time fluorescence quantitative reverse transcription PCR (qRT-PCR) assay, capable of simultaneously detecting DTMUV, DHV, MDRV, and MDPV. This method exhibits high specificity, avoiding cross-reactivity with other viruses such as Fowl adenoviruses (FADV), infectious bursal disease virus (IBDV), infectious bronchitis virus (IBV), infectious laryngotracheitis virus (ILTV), Haemophilus paragallinarum (Hpg), duck circovirus (DUCV), goose astrovirus (GoAstV), and mycoplasma gallisepticum (MG). The limit of detection (LOD) established for DTMUV, DHV, MDRV, and MDPV was determined to be 27 copies/μL. In the repeatability test, the intra-assay and inter-assay coefficients of variation (CVs) of the recombinant plasmid standard were less than 2%. Utilizing this method, we analyzed 326 clinical specimens sourced from Guangxi over the period spanning October 2021 through December 2023, yielding promising and precise outcomes. The qRT-PCR method established herein exhibits commendable specificity, sensitivity, and repeatability. Furthermore, it boasts a high clinical detection rate, making it a highly effective tool for diagnosing these pathogenic agents in waterfowl.

## 1. Introduction

The global panorama of waterfowl viral diseases presents a complicated and dynamic scenario, encompassing a plethora of pathogens that inflict substantial damage upon the waterfowl farming sector. There has been a pronounced escalation in waterfowl viral diseases globally in recent times, featuring outbreaks of duck Tembusu virus (DTMUV), duck hepatitis virus (DHV), Muscovy duck reovirus (MDRV), and Muscovy duck parvovirus (MDPV). After the first large-scale outbreak in China in 2010 [1], DTMUV spread to multiple regions and caused serious economic losses [2]. DHV mainly affects ducklings [3]. Duck virus hepatitis caused by this virus is an infectious disease recorded by the World Organization for Animal Health (OIE), and is of great significance for global public health and animal health. MDRV is an important infectious source of Muscovy duck disease. The virus produces new strains through continuous evolution [4,5], which brings great challenges to its prevention and control. Although MDPV and goose parvovirus (GPV) can infect different hosts, they belong to the family *Parvoviridae* and have certain similarities in virology [6]. They are prone to co-infection and recombination, making it difficult to distinguish them clinically [7,8]. Therefore, it is very important to establish an efficient and practical virus detection method for the detection and prevention of the above waterfowl viral diseases.

DTMUV is a single-stranded positive-sense RNA virus with a gene length of about 10.9 kb, belonging to the *Ntaya* virus group of the genus *Flavivirus* and family *Flaviviridae* [9]. It is an emerging mosquito-borne virus, and its vector is Culex pipiens [10]. In 1955, the virus was first isolated from Culex tritaeniorhynchus in Kuala Lumpur, Malaysia [11]. In 2007, an infectious disease similar to DTMUV was observed in Thailand and then broke out frequently [12]. In 2010, the virus was introduced into China and gradually spread to most duck farming areas [13]. The transmission routes of DTMUV include horizontal transmission and vertical transmission, and its host range is wide. In addition to infecting ducks, it can also infect chickens, geese, mice, pigeons, and houseflies [14,15,16]. It has been reported that DTMUV antibodies have been detected in human serum [17]. The disease mainly invades the ovary, brain, spleen, liver, and other organs, which can cause systemic infection [13,18,19]. It causes nervous system symptoms in ducklings, a sudden drop in egg production and hemorrhagic ovarian inflammation in laying ducks, and testicular atrophy and even testicular interstitial inflammation in male ducks [20,21]. It has been reported that the incidence of the disease is as high as 90%, and the mortality rate is 5–30% [17].

DHV belongs to the family *Picornaviridae*, and is a novel genus *Avihepatovirus* [22], with a gene length of about 7.7 kb [23]. DHV is divided into three serotypes: DHV-1, DHV-2, and DHV-3. DHV-1 was renamed duck hepatitis A virus (DHAV) by the International Committee on Taxonomy of Viruses (ICTV) [24]. DHV was first reported in the United States in 1949 and isolated from chicken embryos the following year [25]. Since then, the virus has been reported in China [26], the United Kingdom [25], America [27], France [28], Australia [29], India [30], South Korea [31], Vietnam [32], and Egypt [33]. DHV mainly infects ducklings within 3 weeks of age, and also infects geese [34]. The virus is mainly transmitted horizontally, mainly through the respiratory tract and digestive tract, but studies have shown that it can also be transmitted vertically [35]. Clinical presentation notably features depression coupled with ataxia [36]. The mortality rate is as high as 80%. It is an acute and highly lethal infectious disease, mainly causing liver enlargement, necrosis, and hemorrhage. Infected ducks can experience varying degrees of functional damage to the liver, brain, spleen, pancreas, and kidneys [37].

MDRV categorically falls within the *Orhtoreovirus* genus of the *Reoviridae* family [38]. The MDRV genome is about 23 kb in length [39]. It is a non-enveloped, icosahedral double-stranded RNA virus with a diameter of 70–80 nm [40]. MDRV was first isolated in France in 1972 [41]. Subsequently, the virus was also found in Israel and Germany [42]. In 1997, a large-scale outbreak of MDRV occurred in China, and in 2000, a new type of Muscovy duck reovirus was isolated in Zhejiang, China [43]. The literature provides evidence of horizontal and vertical transmission of the virus [44]. MDRV demonstrates a diverse host range, including not only ducks but also geese, turkeys, and pigeons [45]. The virus is highly pathogenic to ducklings less than 5 weeks of age [46]. It is capable of decimating the intestinal mucosa and compromising the antioxidant function in ducklings, consequently undermining their mucosal immunity. Infection of ducklings can lead to a large area of white necrosis in the liver and spleen. The clinical manifestations are diarrhea, difficulty in standing, and growth retardation [47]. The morbidity and mortality are high. It has been reported that the mortality rate can reach 60–80% during an outbreak [38].

MDPV is classified as a non-enveloped, single-stranded DNA virus, falling under the genus *dependoparvovirus* within the *Parvoviridae* family [48,49]. The genome of MDPV is approximately 5.1 kb [50]. MDPV was first isolated from France in 1989 [51]. MDPV infection has been reported in China [52], Poland [53], and the United States [51]. MDPV can be transmitted horizontally or vertically [54]. The virus is mainly known as “three weeks disease”, and mainly infects young Muscovy ducklings within 3 weeks of age [55], leading to paralysis and diarrhea [56], and is characterized by ascites, enteritis, myocarditis, and hepatitis [57]. In 2008, a clinical case of duck short beak dwarf syndrome caused by MDPV infection was found in a duck farm in Fujian, China [58]. It has been reported that the virus can cause a mortality rate of 10–80% [59]. MDPV has become a serious pathogen in waterfowl breeding, and has a serious impact on the waterfowl breeding industry.

DTMUV, DHV, MDRV, and MDPV can cause similar clinical symptoms after infection in poultry, such as hepatomegaly and necrosis of poultry, neurological symptoms of standing instability, ataxia, or convulsion. Additionally, DTMUV, DHV, and MDRV infection can each cause spleen lesions in poultry. It is difficult to detect these diseases with similar symptoms in farms, causing huge economic losses to breeding enterprises [60,61]. Therefore, the differential detection of these pathogens through laboratory detection methods is very important for clinical diagnosis. At present, a variety of methods have been established to detect DTMUV [61,62,63], DHV [31,64], MDRV [65,66,67], and MDPV [68,69]. However, qRT-PCR detection methods that can simultaneously identify and detect DTMUV, DHV, MDRV, and MDPV have not been reported. Therefore, this study aims to establish a specific, sensitive, and reproducible one-step multiplex qRT-PCR method for the simultaneous detection and differential diagnosis of DTMUV, DHV, MDRV, and MDPV.

## 2. Materials and Methods

### 2.1. Viruses and Clinical Samples

Professor Meilan Mo donated the infectious bronchitis virus (IBV-M41 strain). DHV (DHAV-SH strain), MDPV (P1 strain), Fowl adenoviruses (FADV-JH strain), infectious bursal disease virus (IBDV-B87 strain), infectious laryngotracheitis virus (ILTV-HN1 strain), Haemophilus paragallinarum (Hpg-HN3 strain), and Mycoplasma gallisepticum (MG-F strain) are preserved in our laboratory. The positive clinical specimens of DTMUV, MDRV, duck circovirus (DUCV), and goose astrovirus (GoAstv) were collected in the field and stored in the laboratory after confirmation by PCR/RT-PCR and gene sequencing.

Between October 2021 and December 2023, 326 clinical specimens were collected from different flocks of ducks at dead animal disposal plants and farms in Guangxi Province. These samples encompassed vital organs such as hearts, livers, spleens, lungs, kidneys, and brains sourced from deceased birds. All clinical samples were stored at −80 °C until use.

### 2.2. Methods

#### 2.2.1. Primers and TaqMan Probes

Based on the genome sequences of DTMUV (GenBank: OQ507679.1), DHV (GenBank: MT157212.1), MDRV (GenBank: GU369968.1), and MDPV (GenBank: ON462352.1), four pairs of specific primers and corresponding TaqMan probes for multiplex qRT-PCR detection were designed using Primer Premier 5 software (Premier, Toronto, ON, Canada). The DTMUV *E* gene amplified a 176 bp fragment, the DHV *3D* gene amplified a 173 bp fragment, the MDRV *S2* gene amplified a 65 bp fragment, and the MDPV *VP1* gene amplified a 70 bp fragment. The sequences of the designed primers and probes were analyzed using the Blast tool of NCBI (https://www.ncbi.nlm.nih.gov/) and the published sequence information to determine the high conservations of primers and probes among different reference strains of DTMUV, DHV, MDRV, and MDPV. The detailed information of primers and probes is shown in Table 1.

#### 2.2.2. Extraction of Nucleic Acid

All viruses and clinical samples were resuspended in phosphate-buffered saline (PBS, pH 7.2) and centrifuged at 4 °C, 12,000× *g*, for 5 min. Total RNA or DNA was extracted from the supernatant using the StarSpin rapid virus DNA/RNA extraction kit (Genstar, Beijing, China), operated according to the manufacturer’s instructions, and stored at −80 °C for use.

#### 2.2.3. Construction of Standard Plasmid

Total RNA was extracted from DTMUV-, DHV-, and MDRV-positive samples by the StarSpin Fast Virus DNA/RNA Kit (Genstar, Beijing, China) and reverse-transcribed into c DNA by StarScript III All-in-one RT Mix with gDNA Remover (Genstar, Beijing, China), and DNA was extracted from MDPV-positive samples. The target gene fragments of the DTMUV *E* gene, DHV *3D* gene, MDRV *S2* gene, and MDPV *VP1* gene were amplified by PCR/RT-PCR by PCR using the c DNA of DTMUV, DHV, and MDRV, and the DNA of MDPV, as templates. The amplified product was purified and cloned into the pMD18-T vector (TaKaRa, Dalian, China) and transformed into *E. coli* DH5α competent cells (TaKaRa, Dalian, China). The positive clones were cultured at 37 °C for 18–20 h, and the plasmid (TaKaRa, Dalian, China) was extracted with MiniBEST Plasmid Purification Kit Ver.4.0 (TaKaRa, Dalian, China) as the plasmid construct. The plasmids were named pDTMUV, pDHV, pMDRV, and pMDPV, respectively, and stored at −80 °C.

The standard plasmid was quantified by UV absorbance at 260 nm and 280 nm using a nanodrop spectrophotometer (Thermo Fisher, Waltham, MA, USA). The exact copy number of the plasmid was calculated using the following formula:

Plasmid copies/µL = 6.02 × 10^23^ × X ng/µL × 10^−9^/plasmid length (bp) × 660

#### 2.2.4. Optimization of Single qRT-PCR Reaction System

The four standard plasmids were mixed at a final concentration of 1:1:1:1, and ten-fold gradient dilution from 2.68 × 10^7^ copies/μL to 2.68 × 10^0^ copies/μL (the final reaction concentration was 2.68 × 10^6^ copies/μL to 2.68 × 10^−1^ copies/μL) to optimize the reaction conditions of DTMUV, DHV, MDRV, and MDPV single qRT-PCR. The reaction mixture contained 10 μL of Premix Ex Taq (Probe qPCR) (2×) (TaKaRa, Dalian, China); DTMUV primers and probes 0.1~0.6 μL (20 pmol/μL), DHV primers and probes 0.1~0.6 μL (20 pmol/μL), MDRV primers and probes 0.1~0.6 μL (20 pmol/μL), and MDPV primers and probes 0.1~0.6 μL (20 pmol/μL); 2.0 μL of plasmid template; and distilled water added to a total of 20 μL. All reactions were amplified by a Pangaea 6 rapid fluorescence quantitative PCR instrument system (Aperbio, Suzhou, China). The amplification procedure included 95 °C pre-deformation for 30 s, 95 °C denaturation for 5 s, annealing at 60 °C for 30 s, and a total of 35~40 cycles. The fluorescence signal was measured at the end of each cycle.

#### 2.2.5. Optimization of Multiplex qRT-PCR Detection

On the basis of determining the optimal reaction conditions of single qRT-PCR, the reaction conditions of multiplex qRT-PCR were further determined by experiments, including primer concentration, probe concentration, and amplification cycle. The reaction mixture contained Premix Ex Taq (Probe qPCR) (2×) (TaKaRa, Dalian, China) 10 μL; DTMUV primers and probes 0.1~0.6 μL (20 pmol/μL), DHV primers and probes 0.1~0.6 μL (20 pmol/μL), MDRV primers and probes 0.1~0.6 μL (20 pmol/μL), and MDPV primers and probes 0.1~0.6 μL (20 pmol/μL); plasmid template 2 μL; and distilled water added to a total of 20 μL. All reactions were amplified by a Pangaea 6 rapid fluorescence quantitative PCR instrument system (Aperbio, Suzhou, China). The amplification procedure included 95 °C pre-deformation for 30 s, 95 °C denaturation for 5 s, annealing at 60 °C for 30 s, and a total of 35~40 cycles. The fluorescence signal was measured at the end of each cycle. The fluorescence signal was measured at the end of each cycle. After amplification, each sample corresponded to a Quantification Cycle value (Cq). Standard plasmids with different dilutions were used as templates to optimize the final concentration of primers and probes, and amplification conditions, to obtain the maximum fluorescence intensity unit (RFU [10^3^]) and the minimum Cq value.

#### 2.2.6. Specificity Analysis of Multiplex qRT-PCR

The specificity of the method was verified by using DNA or RNA of DTMUV, DHV, MDRV, MDPV, FADV, IBDV, IBV, ILTV, Hpg, DUCV, GoAstv, and MG as templates.

#### 2.2.7. Sensitivity Analysis of Multiplex qRT-PCR

The standard plasmids of pDTMUV, pDHV, pMDRV, and pMDPV were mixed at a final concentration of 1:1:1:1, and then ten-fold gradient dilution, from 2.68 × 10^7^ copies/μL to 2.68 × 10^0^ copies/μL (the final reaction concentration was 2.68 × 10^6^ copies/μL to 2.68 × 10^−1^ copies/μL), as multiplex qRT-PCR templates to determine sensitivity.

#### 2.2.8. Repeatability Analysis of Multiplex qRT-PCR

The standard plasmids of pDTMUV, pDHV, pMDRV, and pMDPV were mixed at a final concentration of 1:1:1:1, and 2.68 × 10^6^ copies/μL, 2.68 × 10^4^ copies/μL and 2.68 × 10^2^ copies/μL (final reaction concentrations were 2.68 × 10^5^ copies/μL, 2.68 × 10^3^ copies/μL and 2.68 × 10^1^ copies/μL, respectively) were used as templates to establish multiplex qRT-PCR. The concentrations were tested in triplicate on 3 separate runs. The intra-and inter-assay coefficients of variation (CVs) were measured to evaluate the repeatability of the assay.

#### 2.2.9. Detection of Clinical Samples by Multiplex qRT-PCR

From October 2021 to December 2023, a total of 326 clinical samples were collected from poultry farms in Guangxi Province, southern China. A total of 75 μL of RNA and DNA was extracted from 200 μL of the tissue supernatant using the StarSpin rapid virus DNA/RNA extraction kit (Genstar, Beijing, China) and detected using the developed DTMUV, DHV, MDRV, and MDPV multiplex quantitative qRT-PCR. The PCR or RT-PCR assay standards (http://std.samr.gov.cn (accessed on 18 November 2024)) for DTMUV, DHV, MDRV, and MDPV issued by the Ministry of Agriculture and Rural Affairs of the People’s Republic of China (Standard No. NY/T 3233-2018, Guangxi, China; Standard No. DB34/T 3660-2020, Anhui, China; Standard No. DB34/T3653-2020, Anhui, China; and Standard No. DB35/T 1992-2021, Fujian, China), were used to examine the same clinical samples to compare the assay results of the two methods.

## 3. Results

### 3.1. Construction of Standard Recombinant Plasmid

The results showed that the original concentrations of the four plasmids pDTMUV, pDHV, pMDRV, and pMDPV were 5.08 × 10^11^ copies/μL, 2.68 × 10^11^ copies/μL, 6.41 × 10^11^ copies/μL, and 6.02 × 10^11^ copies/μL, respectively. These plasmids were used as positive standard plasmids to optimize different reaction conditions and the sensitivity and repeatability of multiplex qRT-PCR.

### 3.2. The Optimal Parameters of Multiplex qRT-PCR

After optimization, the reaction conditions were obtained, including denaturation and annealing temperature, primer and probe concentration, and number of amplification cycles. The established multiplex qRT-PCR reaction mixture was as follows: Premix Ex Taq (Probe qPCR) (2×) 10 μL, DTMUV primers and probes 0.4 μL (20 pmol/μL), DHV primers and probes 0.2 μL (20 pmol/μL), MDRV primers and probes 0.6 μL (20 pmol/μL), and MDPV primers and probes 0.5 μL (20 pmol/μL). To total c DNA of 2.0 μL, distilled water was added to a total volume of 20 μL. The amplification procedure was as follows: pre-deformation at 95 °C for 30 s, denaturation at 95 °C for 5 s, and annealing at 60 °C for 30 s, for a total of 40 cycles. The fluorescence signal was measured at the end of each cycle.

### 3.3. Standard Curve of Multiplex qRT-PCR

**Figure 1:**
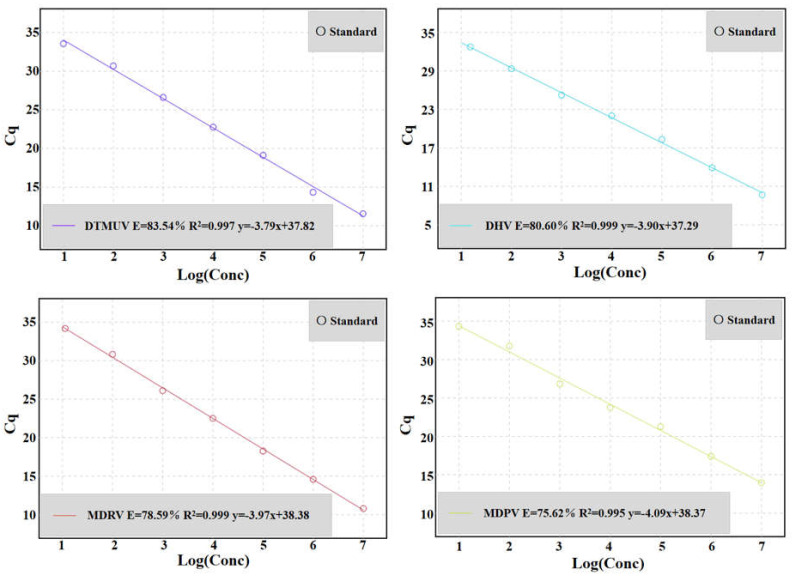
Multiplex qRT-PCR standard curve. Quadratic standard curve showed that there was a linear correlation between the logarithm of copy number and Cq value. The concentration range of standard plasmids (pDTMUV, pDHV, pMDRV, and pMDPV) was 2.68 × 10^7^~2.68 × 10^0^ copies/μL (5.36 × 10^7^~5.36 × 10^0^ copies per reaction).

The standard plasmids of pDTMUV, pDHV, pMDRV, and pMDPV were mixed at a final concentration of 1:1:1:1, and ten-fold gradient dilution to a final concentration of 2.68 × 10^7^~2.68 × 10^0^ copies/μL (5.36 × 10^7^~5.36 × 10^0^ copies per reaction), and a multiplex qRT-PCR standard curve was established. The results showed that the slope of the linear equation of DTMUV was −3.79, the correlation coefficient (R^2^) was 0.997, and the amplification efficiency (E) was 83.54%. The slope of the linear equation of DHV was −3.90, the correlation coefficient (R^2^) was 0.999, and the amplification efficiency (E) was 80.60%. The slope of the linear equation of MDRV was −3.97, the correlation coefficient (R^2^) was 0.999, and the amplification efficiency (E) was 78.59%. The slope of the linear equation of MDPV was −4.09, the correlation coefficient (R^2^) was 0.995, and the amplification efficiency (E) was 75.62% (Figure 1).

The results showed that there was a good linear relationship between the Cq values of the four plasmids after ten-fold gradient dilution, and the linear equation is shown in the figure (R^2^ ≥ 0.995).

### 3.4. Specificity of Multiplex qRT-PCR

**Figure 2:**
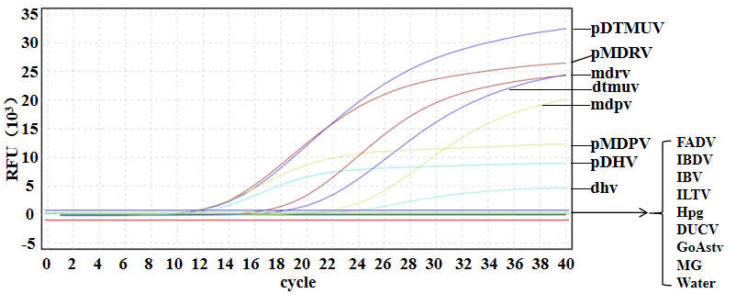
Multiplex qRT-PCR specificity analysis of different virus strains. Standard recombinant plasmids (pDTMUV, pDHV, pMDRV, and pMDPV), DTMUV, DHV, MDRV, MDPV, and other viruses (FADV, IBDV, IBV, ILTV, Hpg, DUCV, GoAstv, MG) were used for specific detection.

In order to evaluate the specificity of the method, RNA/DNA of DTMUV, DHV, MDRV, and MDPV and eight other viruses were detected. Multiplex qRT-PCR was performed using FADV, IBDV, IBV, ILTV, Hpg, DUCV, GoAstv, and MG as templates. The results showed that DTMUV, DHV, MDRV, and MDPV had specific amplification curves, and the other eight viruses had no fluorescence signal or amplification curve, indicating that the detection method had high specificity (Figure 2).

### 3.5. Sensitivity of Multiplex qRT-PCR

**Figure 3:**
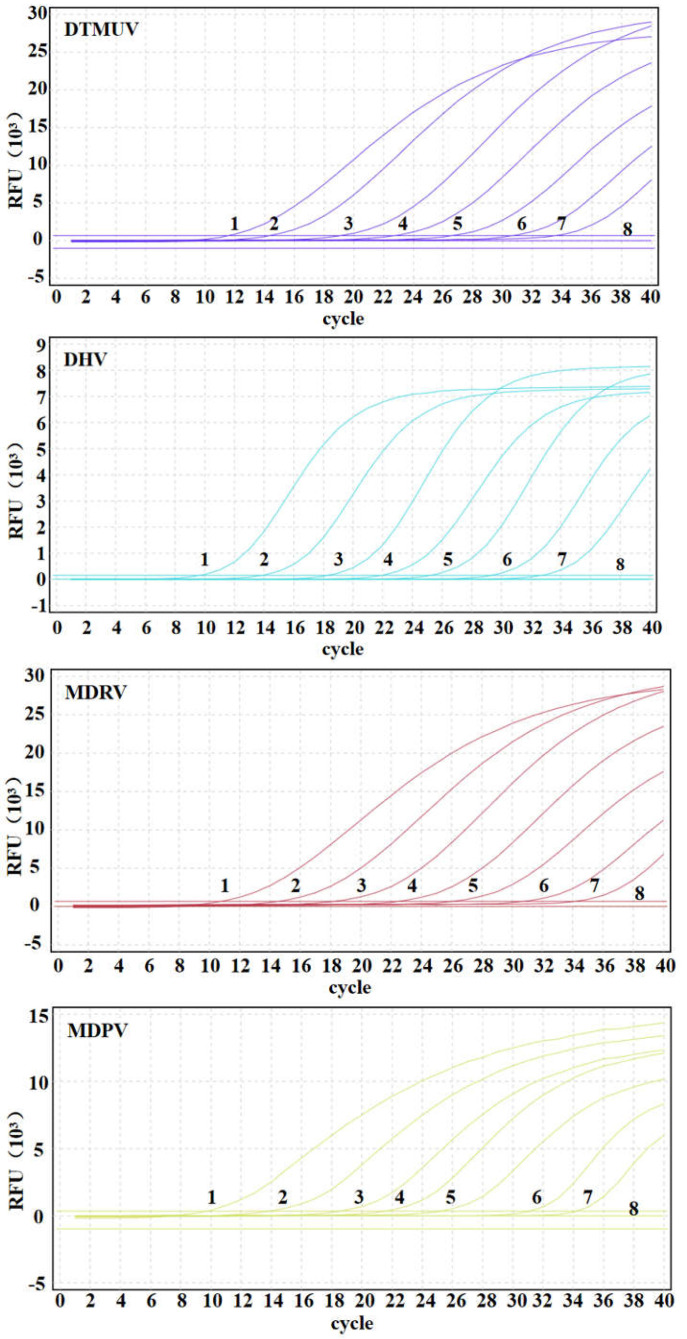
Sensitivity analysis of multiplex qRT-PCR. The sensitivity test was carried out with standard recombinant plasmids (pDTMUV, pDHV, pMDRV, and pMDPV). Curve 1–8: 2.68 × 10^6^~2.68 × 10^−1^ copies/μL (final reaction concentration).

The standard plasmids of pDTMUV, pDHV, pMDRV, and pMDPV were mixed at a final concentration of 1:1:1:1, and ten-fold gradient dilution from 2.68 × 10^7^~2.68 × 10^0^ copies/μL (final reaction concentration: 2.68 × 10^6^ copies/μL~2.68 × 10^−1^ copies/μL) to 2.68 × 10^−1^ copies/μL by 10-fold gradient dilution to detect the sensitivity of multiplex qRT-PCR. The results showed that the limit of detection (LOD) of this method for DTMUV, DHV, MDRV, and MDPV (Figure 3) was 2.68 × 10^1^ copies/μL, indicating that the single qRT-PCR detection had similar sensitivity to multiplex qRT-PCR detection. The Cq values of single and multiplex qRT-PCR are shown in Table 2.

### 3.6. Repeatability of Multiplex qRT-PCR

In order to evaluate the repeatability of the method, the mixed standard plasmids of 2.68 × 10^6^, 2.68 × 10^4^, and 2.68 × 10^2^ copies/μL (final reaction concentration) were used as templates for intra-batch and inter-batch comparison. The results showed that the intra-assay and inter-assay coefficients of variation (CVs) of Cq values were less than 2% (Table 3), indicating that the method had high repeatability.

### 3.7. Multiplex qRT-PCR Detection of Clinical Samples

The established multiplex qRT-PCR method was used to detect 326 clinical samples collected in Guangxi Zhuang Autonomous Region of southern China from October 2021 to December 2023. If the viral load is below the minimum LOD, it is a false negative or inefficient sample collection or harvest, and a secondary test is required for suspected positives within 35–38 Cq The results showed that the positive rates of DTMUV, DHV, MDRV, and MDPV were 6.75% (22/326), 2.15% (7/326), 1.53% (5/326), and 1.84% (6/326), respectively. The co-infection rates of DTMUV and DHV, DHV and MDRV, MDRV and MDPV, and DTMUV and MDPV were 0.61% (2/326), 0.31% (1/326), 0.31% (1/326), and 0.92% (3/326), respectively (Table 4). After the test, all samples were subjected to high-temperature and high-pressure treatment as required. When PCR or RT-PCR detection criteria for DTMUV, DHV, MDRV, and MDPV were used to detect the same clinical samples, the results showed that the detection rates for DTMUV, DHV, MDRV, and MDPV were 6.44% (21/326), 2.15% (7/326), 1.53% (5/326), and 1.53% (5/326), respectively. The co-infection rates of DTMUV and DHV, DHV and MDRV, MDRV and MDPV, and DTMUV and MDPV were 0.61% (2/326), 0.31% (1/326), 0.31% (1/326), and 0.61% (2/326), respectively.

## 4. Discussion

DTMUV, DHV, MDRV, and MDPV are important pathogens in the poultry industry. In avian species, there is a risk of mixed infection. The clinical manifestations of these viral infections are strikingly similar to each other, making visual differentiation at outbreak sites a daunting task that hampers timely pathogen detection efforts. Thus, for the precise diagnosis of the aforementioned ailments, it is necessary to identify these pathogens in the laboratory and obtain clinical information. Compared with ordinary PCR [70,71], qPCR can be used for absolute quantitative or relative quantitative analysis, and can also be used to estimate the relative expression rate of gene expression [72,73]. Among the many diagnostic methods, qRT-PCR is one of the better choices because it can directly detect viral nucleic acids using RNA as a template. This method chooses to use plasmids for gradient dilution, mainly based on the known concentration and copy number of plasmids, and can provide accurate standard curves. Because of its good stability and standardization during storage and operation, the experimental results are more repeatable and reliable. However, plasmids also have certain limitations compared to RNA templates. The target gene sequence in the plasmid is usually artificially cloned. Although it can represent the sequence information of the target gene, compared with natural RNA molecules, the gene sequence on the plasmid may not fully reflect the transcriptional and expression characteristics of the gene in vivo. Therefore, while plasmids are suitable for standardized experiments, they may not fully simulate the true expression of RNA molecules in cells in some cases. However, enzyme-linked immunosorbent assay (ELISA) is also a commonly used molecular biology detection technology, but its quantitative range is limited, there may be cross-reactions, the operation steps are cumbersome, and specific antibodies are required [74,75]. Contrasted with traditional methods like pathogen isolation and identification, qPCR boasts the remarkable capability to swiftly detect a wide array of pathogens in a remarkably brief timeframe. This efficiency makes it particularly advantageous in scenarios requiring rapid response and decision-making [76,77,78]. Multiplex qRT-PCR has high throughput, high sensitivity, and high accuracy. Compared with single qRT-PCR, multiplex qRT-PCR can simultaneously detect multiple pathogens in a short time, and has been widely used in the diagnosis of multiple similar pathogens in laboratories [79,80]. Owing to the reports of mixed infection of these four diseases or serious harm caused by co-occurrence in the same area, these diseases can only be examined one by one, and there is no one-step multi-channel detection method available to quickly distinguish them. Therefore, this study established a one-step multiplex qRT-PCR method for the identification and detection of DTMUV, DHV, MDRV, and MDPV. The method could specifically detect DTMUV, DHV, MDRV, and MDPV. The LOD was 2.68 × 10^1^ copies/μL, and the intra-batch and inter-batch CVs were less than 2%. Li et al. [81] established a double real-time quantitative PCR to detect DTMUV with a minimum LOD of 100 copies/μL, so the detection method established in this experiment has high practicability and good sensitivity. Finally, 326 clinical samples were detected by the established method to further verify its practicability for on-site sample detection.

In this study, specific primers and probes were designed for the DTMUV *E* gene, DHV *3D* gene, MDRV *S2* gene, and MDPV *VP1* gene sequences to amplify the target gene because these gene fragments are relatively conserved in the whole genome sequence of the virus. This can avoid the mutation and recombination of the genes after the mixed infection of the above viruses to a higher extent, and also create favorable conditions for more accurate isolation and identification of these viruses. The *E* gene was expressed stably in the recombinant vaccine [82]. Li et al. pointed out that the truncated E protein was expected to be a potential vaccine to control DTMUV infection in young ducks, which indirectly indicated that its expression had certain stability [83]. Kim et al. realized the differential diagnosis of DHV-1 by amplifying the genes of conserved regions [31]. In order to explore the structure and characteristics of MDRV core protein σ2, Dermody et al. selected the highly conserved nucleotide sequence of the *S2* gene [84]. Yu et al. identified MDPV by amplifying the nucleotide sequence of the *VP1* gene [85], and studies have detected MDPV by amplifying the relatively conserved *VP1* gene sequence [51]. The established qRT-PCR method was used to detect DTMUV, DHV, MDRV, and MDPV in 326 clinical samples from Guangxi, China. The results showed that the positive rates of DTMUV, DHV, MDRV, and MDPV were 6.75%, 2.15%, 1.53%, and 1.84%, respectively. The standard detection methods of PCR or RT-PCR were used to detect the same clinical samples, and the results showed that the detection rates of DTMUV, DHV, MDRV, and MDPV were 6.44%, 2.15%, 1.53%, and 1.53%, respectively. The kappa values of the clinical test results of this method and the clinical test results issued by the Ministry of Agriculture and Rural Affairs of the People’s Republic of China were 0.98, 1, 1, and 0.91. Kappa values were calculated to compare the consistency of the two assays (when K < 0, the consistency strength is very poor; 0–0.2, weak; 0.21–0.4 weak; 0.41–0.6, moderate; 0.61–0.8, high; 0.81–1, very strong). Therefore, it is sufficient to show that the detection method established in this experiment has a high reliability, and the above results also indicate that these viruses are still present in poultry in Guangxi. Given the significant economic toll inflicted upon the poultry industry by DTMUV, DHV, MDRV, and MDPV, it becomes imperative to bolster preventative and control measures against these viruses. By doing so, we can mitigate the financial impact and safeguard the industry’s stability and productivity. Biological safety measures can be strengthened through reasonable site selection, strict management of personnel and vehicles, and maintenance of environmental sanitation. By cultivating well-considered immunization protocols, standardizing vaccine administration procedures, and intensifying preventive initiatives, we can attain a scientifically sound immunization strategy. This approach ensures that immunization efforts are not only effective but also systematic and robust, contributing to the overall health and safety of the profession. The rapid differential diagnosis technology based on this experiment is helpful to strengthen the detection and screening of these duck-derived viruses. In addition, the mixed infection rates of DTMUV and DHV, DHV and MDRV, MDRV and MDPV, and DTMUV and MDPV were 0.61%, 0.31%, 0.31%, and 0.92%, respectively. Although no studies have reported that the above viruses have co-infection data, the results of this experiment show that mixed infection exists. The reason for the low detection rate may be the mixed infection of ducks with different pathogens, which further indicates that DTMUV, DHV, MDRV, and MDPV have a high risk of mixed infection in poultry breeding. For farms, in order to avoid the occurrence of mixed infection, it is necessary to do a good job of daily biosecurity of poultry houses, and strengthen screening and protection, timely vaccination, and close monitoring of symptoms of birds. The qRT-PCR method established in this study has high specificity, sensitivity, repeatability, and practicability. Therefore, the multiplex qRT-PCR method can provide a useful tool for the rapid identification of DTMUV, DHV, MDRV, and MDPV in the clinic of poultry samples suspected of carrying disease.

In this study, the rapid differential detection and diagnosis technology of the above-mentioned diseases was established to accurately detect these duck-borne diseases that have similar symptoms and are difficult to distinguish by clinical and gross examination; provide favorable conditions for improving the detection efficiency of the duck breeding industry; and provide more accurate detection for small and medium-sized duck farms and breeding retail households around the world. The situation provides the necessary technical support for prevention and control work, and is of great significance for promoting the sustainable, stable, and healthy development of the entire duck industry.

## 5. Conclusions

In this study, specific primers and probes were designed according to the sequences of the DTMUV *E* gene, DHV *3D* gene, MDRV *S2* gene, and MDPV *VP1* gene. By optimizing the reaction conditions, such as primer and probe concentrations, annealing temperature, and amplification cycle number, a one-step multiplex qRT-PCR with strong specificity, high sensitivity, and good repeatability was successfully established for simultaneous detection and differential diagnosis of DTMUV, DHV, MDRV, and MDPV.

## Figures and Tables

**Table 1:** Primers and probes for detection of DTMUV, DHV, MDRV, and MDPV.

Primers and Probes	Sequence (5′→3′)	Concentration (μM)	Product Size (bp)
DTMUV-F	AAGCTTTCACGTCAACAC	10	176
DTMUV-R	CATGCCTTGAGTAATCCACGA	10	
DTMUV-Q	ACTGAGCCAAAATCCCATGC	10	
DHV-F	ACTTTTCTGGTTTTGACGG	10	173
DHV-R	TGAGCACATACCACCTTC	10	
DHV-Q	TTCACAAGGGCTGGATCGTT	10	
MDRV-F	CCCAATGTTGTGGCGTTCTA	10	65
MDRV-R	ATGGTGCGGGAAGCAAAC	10	
MDRV-Q	ATTATGGCGCGCCTCCAACGG	10	
MDPV-F	TTTACGGATGACGAGCATCAAC	10	70
MDPV-R	GGAACGGCGGCATGGT	10	
MDPV-Q	CCCGTATGTCCTGGGCTCGGC	10	

**Table 2:** Comparison of the Cq values between the singleplex and multiplex qRT-PCR.

Plasmid	Concentration (Copies/μL)	2.68 × 10^6^	2.68 × 10^5^	2.68 × 10^4^	2.68 × 10^3^	2.68 × 10^2^	2.68 × 10^1^	2.68 × 10^0^	2.68 × 10^−1^
DTMUV	Singleplex qRT-PCR	11.38	13.89	19.02	21.93	26.72	31.06	33.17	(none)
	Multiplex qRT-PCR	11.56	14.33	19.12	22.76	26.60	30.68	33.55	(none)
DHV	Singleplex qRT-PCR	9.78	13.47	17.19	21.34	24.67	30.01	31.93	(none)
	Multiplex qRT-PCR	9.69	13.90	18.31	22.00	25.24	29.34	32.74	(none)
MDRV	Singleplex qRT-PCR	10.49	15.19	18.43	22.61	27.07	31.23	35.02	(none)
	Multiplex qRT-PCR	10.80	14.58	18.27	22.51	26.11	30.83	34.19	(none)
MDPV	Singleplex qRT-PCR	9.17	13.43	17.61	21.54	25.18	30.50	33.48	(none)
	Multiplex qRT-PCR	9.75	13.86	18.50	21.50	25.21	31.11	34.18	(none)

**Table 3:** Repeatability analysis of multiplex qRT-PCR for Cq values.

Plasmid	Concentration (Copies/μL)	Cq Values of Intra-Assay			Cq Value of Inter-Asssay		
		$\bar{X}$	SD	CV (%)	$\bar{X}$	SD	CV (%)
DTMUV	2.68 × 10^6^	11.29	0.36	1.34	11.31	0.28	1.36
	2.68 × 10^4^	19.45	0.15	0.96	19.54	0.16	1.23
	2.68 × 10^2^	26.69	0.18	0.83	26.73	0.20	0.78
DHV	2.68 × 10^6^	9.82	0.24	1.41	9.76	0.15	1.21
	2.68 × 10^4^	17.63	0.17	0.68	17.59	0.16	0.93
	2.68 × 10^2^	24.84	0.15	0.72	24.91	0.15	0.98
MDRV	2.68 × 10^6^	10.58	0.16	1.37	10.62	0.23	1.27
	2.68 × 10^4^	18.52	0.14	1.04	18.47	0.18	1.32
	2.68 × 10^2^	26.37	0.25	0.81	26.32	0.25	0.98
MDPV	2.68 × 10^6^	9.09	0.21	1.28	8.95	0.13	0.84
	2.68 × 10^4^	18.70	0.18	0.79	18.64	0.16	1.15
	2.68 × 10^2^	25.36	0.15	0.87	25.47	0.15	0.63

**Table 4:** Multiplex qRT-PCR detection of clinical samples.

Date	Numbers	DTMUV (%)	DHV (%)	MDRV (%)	MDPV (%)	DTMUV + DHV (%)	DHV + MDRV (%)	MDRV + MDPV (%)	DTMUV + MDPV (%)
October 2021	12	1 (8.33)	1 (8.33)	0 (0)	0 (0)	1 (8.33)	0 (0)	0 (0)	0 (0)
November 2021	10	0 (0)	0 (0)	0 (0)	1 (10.00)	0 (0)	0 (0)	0 (0)	0 (0)
January 2022	9	1 (11.11)	0 (0)	0 (0)	0 (0)	0 (0)	0 (0)	0 (0)	0 (0)
April 2022	27	2 (7.40)	1 (3.70)	0 (0)	0 (0)	0 (0)	0 (0)	0 (0)	0 (0)
May 2022	39	5 (12.82)	1 (2.56)	2 (5.13)	1 (2.56)	1 (2.56)	0 (0)	0 (0)	0 (0)
October 2022	35	3 (8.57)	0 (0)	0 (0)	0 (0)	0 (0)	0 (0)	1 (2.86)	0 (0)
November 2022	20	0 (0)	1 (5.00)	0 (0)	0 (0)	0 (0)	0 (0)	0 (0)	0 (0)
December 2022	34	2 (5.89)	0 (0)	0 (0)	3 (8.82)	0 (0)	0 (0)	0 (0)	1 (2.94)
January 2023	26	2 (7.69)	0 (0)	1 (3.85)	0 (0)	0 (0)	0 (0)	0 (0)	0 (0)
March 2023	21	1 (4.76)	0 (0)	0 (0)	0 (0)	0 (0)	0 (0)	0 (0)	0 (0)
April 2023	19	0 (0)	2 (10.53)	0 (0)	0 (0)	0 (0)	0 (0)	0 (0)	0 (0)
September 2023	16	0 (0)	1 (6.25)	0 (0)	0 (0)	0 (0)	0 (0)	0 (0)	0 (0)
October 2023	28	2 (7.14)	0 (0)	0 (0)	0 (0)	0 (0)	1 (3.57)	0 (0)	0 (0)
December 2023	30	3 (10.00)	0 (0)	2 (6.67)	1 (3.33)	0 (0)	0 (0)	0 (0)	2 (6.67)
Total	326	22 (6.75)	7 (2.15)	5 (1.53)	6 (1.84)	2 (0.61)	1 (0.31)	1 (0.31)	3 (0.92)

## Data Availability

The original contributions presented in the study are included in the article and Supplementary Materials, further inquiries can be directed to the corresponding author.

## Supplementary Materials

The following supporting information can be downloaded at: https://www.mdpi.com/article/10.3390/microorganisms12122423/s1, Table S1: Statistical table of 326 poultry samples in Guangxi. https://kdocs.cn/l/ciBXRy4NMIIe (accessed on 18 November 2024).
